# Evaluation of the Efficacy of Stem Cell Therapy in Ovariectomized Osteoporotic Rats Based on Micro-CT and Dual-Energy X-Ray Absorptiometry: A Systematic Review and Meta-Analysis

**DOI:** 10.1155/2021/1439563

**Published:** 2021-08-10

**Authors:** Zhencheng Xiong, Ping Yi, Jialiang Lin, Shengfeng Qiu, Li Shu, Chi Zhang

**Affiliations:** ^1^Institute of Medical Technology, Peking University Health Science Center, Beijing 100191, China; ^2^Peking University Third Hospital, Beijing 100191, China; ^3^Department of Spine Surgery, China-Japan Friendship Hospital, Beijing 100029, China; ^4^Department of Orthopedics, Peking University Third Hospital, Beijing 100191, China; ^5^Department of Obstetrics and Gynecology, Xingguo County People's Hospital, Ganzhou, Jiangxi 342400, China; ^6^Department of Orthopedics, Peking University International Hospital, Beijing 102206, China; ^7^Biomedical Engineering Department, Peking University, Beijing 100191, China

## Abstract

**Objective:**

Osteoporosis is an abnormal bone metabolism disease characterized by microstructural degeneration of bone tissue and reduction in bone mass, resulting in increased brittleness of bone tissue and susceptibility to fracture. Due to the tissue regenerative potential of stem cell transplantation, it is now used in the treatment of various disease models such as osteoporosis. The purpose of this work is to carry out a systematic review and meta-analysis of the efficacy of stem cell therapy in ovariectomized (OVX) osteoporotic rats.

**Methods:**

PubMed, Cochrane Library, ScienceDirect, Embase, CNKI, and Wanfang Databases were used to search for articles that met the inclusion criteria. Two researchers independently screened the articles that met the inclusion criteria. RevMan 5.3 and STATA 16.0 were used for data analysis. This meta-analysis was registered at INPLASY with reference number ID: INPLASY202150017.

**Results:**

Thirteen eligible studies were selected, including 405 rats. The sources of stem cells are divided into four main categories: bone marrow mesenchymal stem cells (BMSCs), adipose-derived stem cells (ADSCs), amniotic membrane mesenchymal stem cells (AM-MSCs), and human umbilical cord blood-derived mesenchymal stem cells (hUCB-MSCs). Compared with the OVX group, both stem cell transplantation groups had higher bone mineral density (BMD) (BMSCs: SMD = 2.01, 95% CI: [1.38, 2.63], *P* < 0.001, *I*^2^ = 76.6%; ADSCs: SMD = 2.24, 95% CI: [0.79, 3.69], *P* = 0.003, *I*^2^ = 86.7%) and bone volume/total volume (BV/TV) (hUCB-MSCs: SMD = 1.71, 95% CI: [0.97, 2.44], *P* < 0.001, *I*^2^ = 0%; ADSCs: SMD = 2.16, 95% CI: [0.27, 4.04], *P* = 0.025, *I*^2^ = 82.6%). In the BMSC treatment groups, the trabecular numbers (Tb.N) (SMD = 4.28, 95% CI: [0.91, 7.64], *P* = 0.013, *I*^2^ = 94.9%) were significantly higher, whereas the results for trabecular thickness (Tb.Th) (SMD = 2.7, 95% CI: [-0.34, 5.73], *P* = 0.081, *I*^2^ = 95.4%) and trabecular spacing (Tb.Sp) (SMD = −3.08, 95% CI: [-6.55, 0.38], *P* = 0.081, *I*^2^ = 96.3%) were not statistically significant compared to those of the OVX group. The stem cell transplantation group had a low BMD, BV/TV, and Tb.N compared to the sham operation group.

**Conclusion:**

Stem cell therapy may increase bone strength, bone volume, and the number of trabeculae in OVX osteoporotic rats. The results of this meta-analysis showed the potential therapeutic effect of stem cell transplantation in OVX osteoporotic rats, bringing new therapeutic ideas and directions to the clinical treatment of osteoporosis. Due to the limited number and quality of studies related to some outcomes, more high-quality RCTs are still needed in the future to complement the existing findings.

## 1. Introduction

Osteoporosis is a metabolic bone disease characterized by low bone mass and destruction of bone tissue microarchitecture, leading to increased bone fragility and fracture risk in patients [1]. Osteoporosis is more common in the elderly, especially in postmenopausal women [2]. As the average life expectancy increases, more and more countries are entering an aging society, and the social burden caused by the increase in the incidence of osteoporosis is becoming more and more serious [3]. Deficiency of estrogen after menopause usually leads to the development of osteoporosis. The ovariectomized (OVX) rat model provides us with a suitable model to examine the mechanism of osteoporosis [4]. Because osteoporosis is usually an imbalance of bone resorption and bone formation caused by estrogen deficiency or aging, certain pharmacological agents, such as those that promote bone formation (parathyroid hormone) and those that inhibit osteoclast resorption (bisphosphonates), are widely used in the treatment of osteoporosis [4]. However, with the widespread clinical use of these drugs, adverse side effects have also been observed, such as drug-related osteonecrosis of the jaw [5]. Therefore, the search for new osteoporosis treatment strategies is of great clinical importance.

In recent years, tissue engineering technology has been rapidly developed in the fields of bone and cartilage tissue construction, tendon ligaments, blood vessels, nerves, skin, and oral tissues [6]. Stem cells, an important component of tissue engineering technology, have received a lot of attention due to their potential capabilities [7]. Stem cells, a class of undifferentiated or partially differentiated cells with unlimited self-proliferative and multidirectional differentiation capabilities, have been shown to be closely associated with the progression of osteoporosis [7]. Stem cell transplantation is proposed as a potential treatment strategy for patients with osteoporosis [8]. Some experimental studies have been conducted in animal models of osteoporosis to evaluate the therapeutic effect of stem cell transplantation [8–21]. Stem cells used in animal models of osteoporosis include bone marrow mesenchymal stem cells (BMSCs) [21], adipose-derived stem cells (ADSCs) [16], amniotic membrane mesenchymal stem cells (AM-MSCs) [12], and human umbilical cord blood-derived mesenchymal stem cells (hUCB-MSCs) [20]. Among them, BMSCs are able to differentiate into multiple cell types, including osteoblasts, chondrocytes, and adipocytes, under appropriate culture conditions and are the most commonly used MSC for osteoporosis due to their easy accessibility and strong osteogenic differentiation [4, 19]. Some of the current experimental studies evaluated the potential therapeutic effects of stem cell transplantation in OVX osteoporotic rats by using micro-CT and dual-energy X-ray absorptiometry [9–21]. In order to investigate the potential efficacy of stem cell transplantation in OVX osteoporotic rats and thus provide some support for the possibility of stem cell transplantation in the treatment of osteoporosis, we constructed this meta-analysis by pooling the relevant studies mentioned above.

## 2. Materials and Methods

### 2.1. Search Method

After identifying the topics for this meta-analysis, in order to obtain all relevant studies, two researchers from our research team each independently searched multiple databases according to the Cochrane Collaboration guidelines, including PubMed (1966 to April 1, 2021), Cochrane Library (1966 to April 1, 2021), ScienceDirect (1980 to April 1, 2021), Embase (1980 to April 1, 2021), CNKI (1980 to April 1, 2021), and Wanfang Databases (1980 to April 1, 2021). Literature search is achieved by concatenating MeSH terms and corresponding keywords using Boolean operators (AND or OR), including “stem cell,” “mesenchymal stem cell or MSC,” “bone marrow-derived mesenchymal stem cell or BMSC,” “adipose-derived stem cell or ADSC,” “osteoporosis,” “ovariectomized or OVX,” and “rat.” Two researchers independently screened all the retrieved articles, first one by one, based on title and abstract, and then later on for full-text detailed reading. Finally, additional screening of relevant studies is performed based on the references of the identified included studies. The two lists of literature obtained above will be discussed in our team to integrate and resolve differences. The Preferred Reporting Items for Systematic Reviews and Meta-Analyses (PRISMA) statement is an indispensable reference for the current meta-analysis [22].

### 2.2. Study Screening

All retrieved articles were screened by our research team according to the inclusion and exclusion criteria developed by the subject of this meta-analysis. Inclusion criteria included the following: (1) all studies involved comparing the effects of stem cell therapy to OVX control group or sham-operated group, (2) all included studies could be of either randomized controlled trial (RCT) or non-RCT, (3) the animal model was rat and osteoporosis model establishment was achieved by ovariectomy, (4) the source of stem cells was not limited, (5) the data of outcome measurements were obtained by micro-CT or dual-energy X-ray absorptiometry, and (6) the data related to the outcome measurements could be successfully extracted.

Exclusion criteria included the following: (1) the study lacked a control group that met the inclusion criteria; (2) the animal model was a mouse, rabbit, or other experimental animals; (3) the osteoporosis model was not established by ovariectomy; (4) the data related to outcome measurements could not be extracted; (5) the study type was a review, conference abstract, commentary, case report, or letter; and (6) all studies that did not meet the inclusion criteria.

### 2.3. Required Data Extraction

The extraction of the required data was done independently by two researchers, and then, another researcher aggregated the data and resolved the divergent data after discussion within the research team. The main data extracted for this meta-analysis were the results of micro-CT and dual-energy X-ray absorptiometry, where bone mineral density (BMD) was the primary outcome measurement, and bone volume/total volume (BV/TV), trabecular number (Tb.N), trabecular thickness (Tb.Th), and trabecular spacing (Tb.Sp) were the secondary outcome measurement. We also extracted the following data: first author, year of publication, country/region, study type, number of rats (experimental group : control group), rat type, rat month age, sex, surgical method, and intervention (experimental group : control group).

### 2.4. Quality Assessment of Included Studies

The quality of RCTs in meta-analyses was usually assessed according to the Cochrane Handbook for Systematic Reviews [23]. Two researchers independently used RevMan software to create a “risk of bias” table with seven main elements to assess the quality of each included RCT. Each element could be judged as one of high risk of bias, low risk of bias, or unclear risk of bias based on the actual content of the study.

The Newcastle-Ottawa Scale (NOS) containing three main items (selection, comparability, and outcome) can be used to assess the quality of the included non-RCTs [24]. This scale is also subdivided into eight detailed quality items (selection: 4 quality items; comparability: 1 quality item; outcome: 3 quality items). In “selection” and “outcome,” each item can be awarded up to one star; in “comparability”, a maximum of two stars can be given to a unique item. The more stars a study receives, the higher the quality assessment. Low quality (0-3), medium quality (4-6), and high quality (7-9) studies each have a range of scores.

### 2.5. Statistical Analysis

Outcome measurements were analyzed in subgroups according to stem cell source or bone trabecula assessment methods. Because the included outcome measurements were continuous data, as well as unit differences, we used standard mean difference (SMD) and 95% confidence interval (CI) for the analysis. Heterogeneity of the included studies was assessed by *I*^2^, which was considered as low, moderate, and high heterogeneity when the value of *I*^2^ was 25%, 50%, and 75%, respectively [25]. The choice of the random effect model and fixed effect model is determined by *I*^2^, and the former is executed when *I*^2^ > 50% and *P* < 0.1. Otherwise, the latter is executed. Statistical analysis of all data was performed by using STATA software version 16.0 and RevMan 5.3 for Windows. In this meta-analysis, the results were considered statistically significant when *P* < 0.05.

## 3. Results

### 3.1. Search Results from Literature

**Figure 1:**
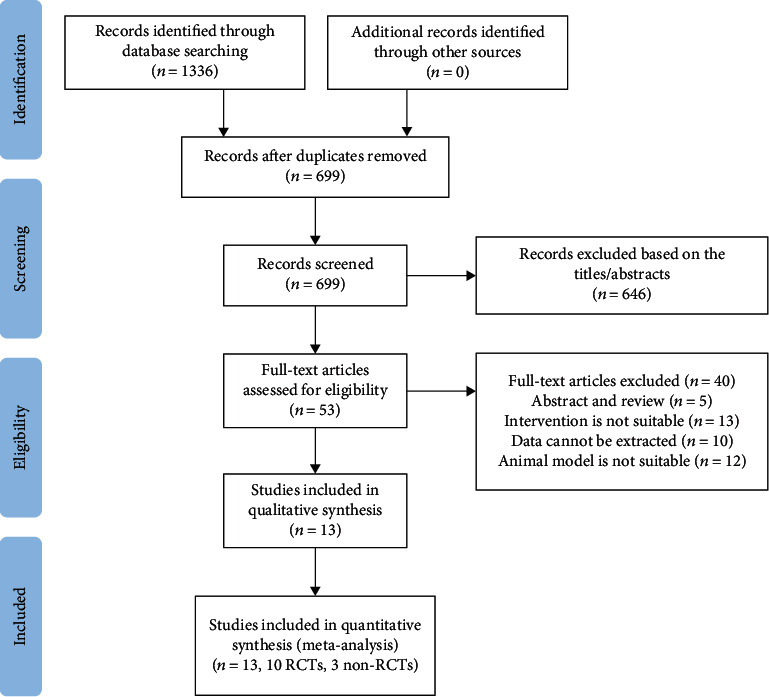
Flow chart of literature search and screening for meta-analysis.

Based on the search strategy and inclusion and exclusion criteria, a total of 1,336 potentially relevant articles were generated, including from PubMed (*n* = 494), Cochrane Library (*n* = 17), ScienceDirect (*n* = 468), Embase (*n* = 88), CNKI (*n* = 207), and Wanfang Databases (*n* = 62). After two researchers carefully screened the titles and abstracts independently, and briefly reviewed the full text, a total of 646 articles were excluded. Then, according to the inclusion and exclusion criteria, a detailed evaluation was carried out for the full text of the remaining 53 articles. Finally, the meta-analysis included 10 RCTs and 3 non-RCTs (Figure 1) [9–21].

### 3.2. Characteristics of the Included Studies

A total of 10 RCTs and 3 non-RCTs involving 405 rats were included in this meta-analysis, all published from 2008 to 2020 [9–21]. All included studies explored the efficacy of stem cell transplantation in OVX osteoporotic rats. There are 4 sources of stem cells in the included studies, of which 8 studies are BMSCs [9, 10, 13, 14, 17–19, 21], 3 studies are ADSCs [11, 15, 16], one study is AM-MSCs [12], and the remaining 1 study is hUCB-MSCs [20]. Female rats were used in all 13 included studies, 11 of which were Sprague Dawley (SD) rats [9–11, 13–18, 20, 21] and 2 of which were Wistar rats [12, 19]. The establishment of osteoporosis models was all achieved by ovariectomy. Of the 13 studies, 10 included an OVX blank control group and a sham-operated group [9–15, 18–20], 2 studies included only an OVX control group [16, 17], and one study remained that included only a sham-operated group [21]. In the OVX blank control group, only ovariectomy was performed, and no stem cell transplantation was performed; in the sham-operated group, no ovariectomy or stem cell transplantation was performed, and only the skin was cut and sutured. The method of stem cell transplantation also varies, with most studies involving the injection of stem cells via the tail vein and others involving direct injection into the femur or tibia. The site of tissue sampling varied, with eight studies sampling only the femur [10–12, 16, 17, 19–21], two studies sampling only the tibia [13, 18], and three studies sampling the femur and the lumbar vertebrae [9, 14, 15]. One study was a combination of AM-MSCs and zoledronic acid [12], another study was a combination of ADSCs and icariin [15], and finally, another study was BMSCs cocultured with nano-HA, Pt-NPs, or Pt-HA-nanocomposite [19]. The 13 included studies were also not entirely consistent in the injection doses of stem cells and the timing of specimens taken after injection.  Table 1 lists the characteristics of all included studies.

### 3.3. Assessment of the Risk of Bias in the Included Studies

**Figure 2:**
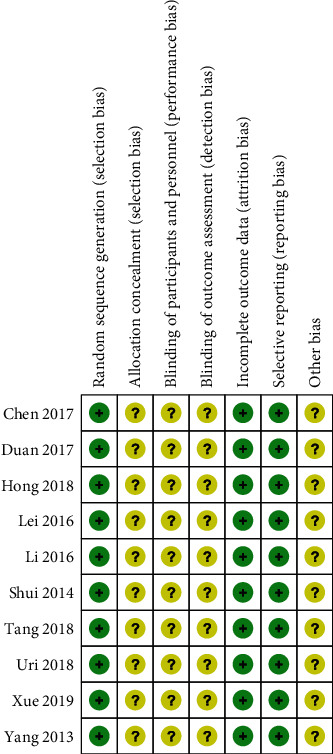
Risk of bias summary. +: low risk of bias; −: high risk of bias; ?: bias unclear.

The risk of bias assessment for the 10 included RCTs is displayed in Figure 2. The included studies were all animal studies, none of the 10 studies explicitly mentioned blinding and allocation concealment, but all stated random assignment [9–16, 18, 20]. No selective reporting or incomplete outcome data were found. Other biases could not be accurately determined.

The risk of bias assessment for these 3 non-RCTs is displayed in Table 2. Item-by-item scoring of the 3 studies according to the NOS showed that 2 studies [17, 21] received a score of 6 and the remaining one [19] a score of 7, indicating that the quality of the included studies was acceptable.

### 3.4. Results of the Meta-Analysis

#### 3.4.1. BMD

**Figure 3:**
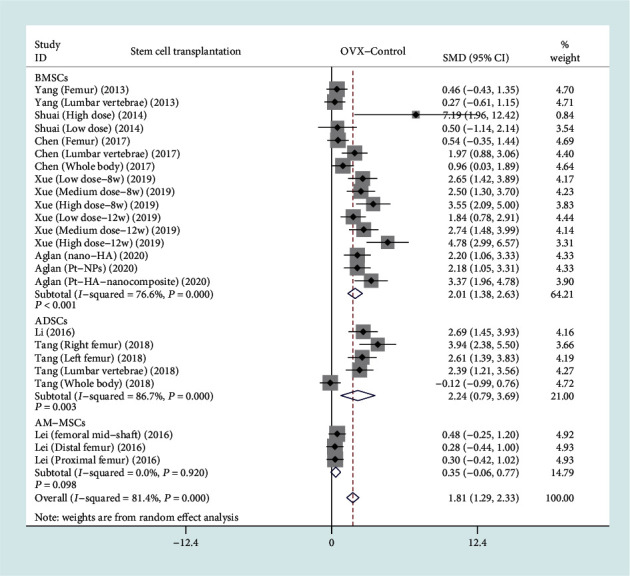
Forest plot showing the effect of stem cell treatment group compared with OVX control group on BMD. OVX: ovariectomized; BMD: bone mineral density; BMSCs: bone marrow mesenchymal stem cells; ADSCs: adipose-derived stem cells; AM-MSCs: amniotic membrane mesenchymal stem cells; SMD: standard mean difference.

Among the 13 included studies, there are a total of 9 studies with BMD as the primary outcome measurement [9–12, 14, 15, 18, 19, 21]. According to the difference of the control group, we conducted a comparative analysis of the stem cell treatment group and the OVX control group, as well as the stem cell treatment group and the sham operation group. The forest plot in Figure 3 shows the effect of the stem cell treatment group on BMD compared to the OVX control group, with a total of eight studies included [9–12, 14, 15, 18, 19]. BMD was divided into 3 subgroups based on different stem cell sources. A total of five studies (128 rats) provided BMD data after BMSC transplantation [9, 10, 14, 18, 19], two studies (40 rats) provided BMD data after ADSC transplantation [11, 15], and one study (30 rats) provided BMD data after AM-MSC transplantation [12]. In view of the significant heterogeneity (*I*^2^ > 50%, *P* < 0.1), we used a random effect model. There was a statistically significant difference on BMD between the BMSC transplantation group and the OVX control group based on the results of the pooled analysis (SMD = 2.01, 95% CI: [1.38, 2.63], *P* < 0.001, *I*^2^ = 76.6%), and there was a statistically significant difference between the ADSC transplantation and the OVX control group (SMD = 2.24, 95% CI: [0.79, 3.69], *P* = 0.003, *I*^2^ = 86.7%). There is only one study on AM-MSC transplantation, and the results showed no statistically significant differences (SMD = 0.35, 95% CI: [-0.06, 0.77], *P* = 0.098, *I*^2^ = 0%).

**Figure 4:**
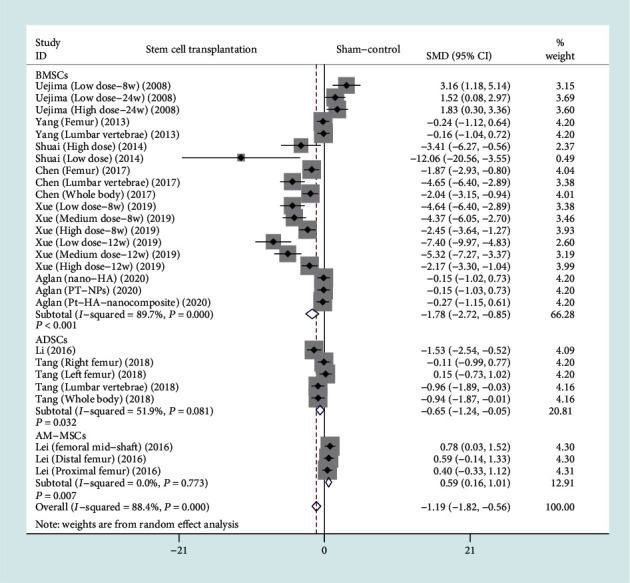
Forest plot showing the effect of stem cell treatment group compared with sham operation group on BMD. BMD: bone mineral density; BMSCs: bone marrow mesenchymal stem cells; ADSCs: adipose-derived stem cells; AM-MSCs: amniotic membrane mesenchymal stem cells.

The forest plot in Figure 4 shows the effect of the stem cell treatment group on BMD compared to the sham operation group, with a total of nine studies included [9–12, 14, 15, 18, 19, 21]. BMD was divided into 3 subgroups based on different stem cell sources. A total of six studies (148 rats) provided BMD data after BMSC transplantation [9, 10, 14, 18, 19, 21], two studies (40 rats) provided BMD data after ADSC transplantation [11, 15], and one study (30 rats) provided BMD data after AM-MSC transplantation [12]. In view of the significant heterogeneity (*I*^2^ > 50%, *P* < 0.1), we used a random effect model. There was a statistically significant difference on BMD between the BMSC transplantation group and the sham operation group based on the results of the pooled analysis (SMD = −1.78, 95% CI: [-2.72, -0.85], *P* < 0.001, *I*^2^ = 89.7%), and there was a statistically significant difference between the ADSC transplantation and the sham operation group (SMD = −0.65, 95% CI: [-1.24, -0.05], *P* = 0.032, *I*^2^ = 51.9%), and there was a statistically significant difference between the AM-MSC transplantation and the sham operation group (SMD = 0.59, 95% CI: [0.16, 1.01], *P* = 0.007, *I*^2^ = 0%).

#### 3.4.2. BV/TV

**Figure 5:**
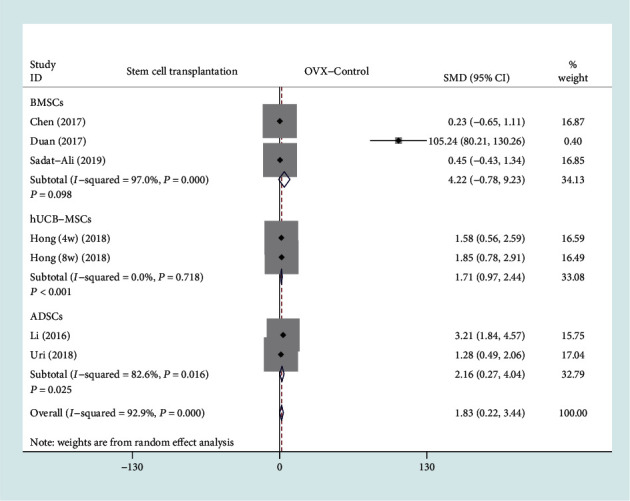
Forest plot showing the effect of stem cell treatment group compared with OVX control group on BV/TV. BV/TV: bone volume/total volume; BMSCs: bone marrow mesenchymal stem cells; hUCB-MSCs: human umbilical cord blood-derived mesenchymal stem cells; ADSCs: adipose-derived stem cells.

Among the 13 included studies, there are a total of 6 studies with BV/TV as the secondary outcome measurement [11, 13, 14, 16, 17, 20]. The forest plot in Figure 5 shows the effect of the stem cell treatment group on BV/TV compared to the OVX control group, with a total of six studies included. BV/TV was divided into 3 subgroups based on different stem cell sources. A total of three studies (58 rats) provided BV/TV data after BMSC transplantation [13, 14, 17], two studies (50 rats) provided BV/TV data after ADSC transplantation [11, 16], and one study (20 patients) provided BV/TV data after hUCB-MSC transplantation [20]. In view of the significant heterogeneity (*I*^2^ > 50%, *P* < 0.1), we used a random effect model. There was a statistically significant difference on BV/TV between the ADSC transplantation group and the OVX control group based on the results of the pooled analysis (SMD = 2.16, 95% CI: [0.27, 4.04], *P* = 0.025, *I*^2^ = 82.6%), and there was a statistically significant difference between the hUCB-MSC transplantation and the OVX control group (SMD = 1.71, 95% CI: [0.97, 2.44], *P* < 0.001, *I*^2^ = 0%). However, there were no statistically significant differences between the BMSC transplantation group and the OVX control group (SMD = 4.22, 95% CI: [-0.78, 9.23], *P* = 0.098, *I*^2^ = 97%).

**Figure 6:**
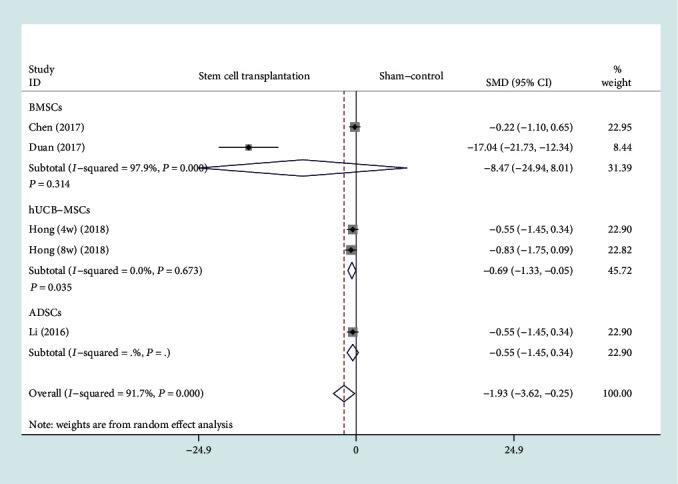
Forest plot showing the effect of stem cell treatment group compared with sham operation group on BV/TV. BV/TV: bone volume/total volume; BMSCs: bone marrow mesenchymal stem cells; hUCB-MSCs: human umbilical cord blood-derived mesenchymal stem cells; ADSCs: adipose-derived stem cells.

The forest plot in Figure 6 shows the effect of the stem cell treatment group on BV/TV compared to the sham operation group, with a total of four studies included [11, 13, 14, 20]. BV/TV was divided into 3 subgroups based on different stem cell sources. A total of two studies (48 rats) provided BV/TV data after BMSC transplantation [13, 14], one study (20 rats) provided BV/TV data after ADSC transplantation [11], and one study (20 patients) provided BV/TV data after hUCB-MSC transplantation [20]. In view of the significant heterogeneity (*I*^2^ > 50%, *P* < 0.1), we used a random effect model. There was a statistically significant difference between the hUCB-MSC transplantation and the sham operation group (SMD = −0.69, 95% CI: [-1.33, -0.05], *P* = 0.035, *I*^2^ = 0%). However, there were no statistically significant differences between the BMSC transplantation group and the sham operation group (SMD = −8.47, 95% CI: [-24.94, 8.01], *P* = 0.314, *I*^2^ = 97.9%). Because there is only one set of data that provides data on BV/TV between the ADSC transplantation group and the sham operation group, no conclusion can be drawn.

#### 3.4.3. Tb.N, Tb.Th, and Tb.Sp

**Figure 7:**
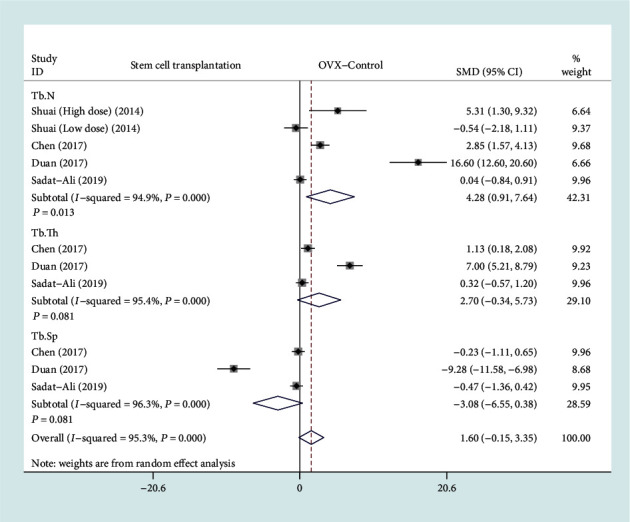
Forest plot showing the effect of the BMSC transplantation group compared with the OVX control group on Tb.N, Tb.Th, and Tb.Sp. Tb.N: trabecular number; Tb.Th: trabecular thickness; Tb.Sp: trabecular spacing.

Among the 13 included studies, there are a total of 4 studies with Tb.N, Tb.Th, and Tb.Sp as the secondary outcome measurement [10, 13, 14, 17]. The forest plot in Figure 7 shows the effect of the BMSC transplantation group on Tb.N, Tb.Th, and Tb.Sp compared to the OVX control group, with a total of four studies included. A total of four studies (67 rats) provided Tb.N data [10, 13, 14, 17], and three studies (58 rats) provided Tb.Th and Tb.Sp data [13, 14, 17]. In view of the significant heterogeneity (*I*^2^ > 50%, *P* < 0.1), we used a random effect model. There was a statistically significant difference on Tb.N between the BMSC transplantation group and the OVX control group based on the results of the pooled analysis (SMD = 4.28, 95% CI: [0.91, 7.64], *P* = 0.013, *I*^2^ = 94.9%). However, there were no statistically significant differences on Tb.Th and Tb.Sp between the BMSC transplantation group and the OVX control group (Tb.Th: SMD = 2.7, 95% CI: [-0.34, 5.73], *P* = 0.081, *I*^2^ = 95.4%; Tb.Sp: SMD = −3.08, 95% CI: [-6.55, 0.38], *P* = 0.081, *I*^2^ = 96.3%).

**Figure 8:**
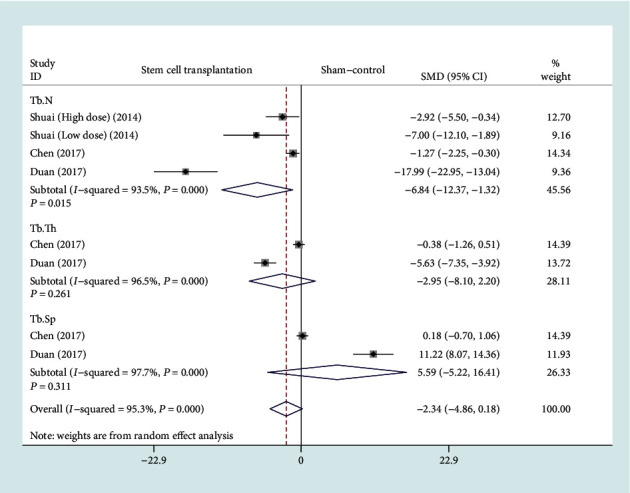
Forest plot showing the effect of the BMSC transplantation group compared with the sham operation group on Tb.N, Tb.Th, and Tb.Sp. Tb.N: trabecular number; Tb.Th: trabecular thickness; Tb.Sp: trabecular spacing.

The forest plot in Figure 8 shows the effect of the BMSC transplantation group on Tb.N, Tb.Th, and Tb.Sp compared to the sham operation group, with a total of three studies included [10, 13, 14]. A total of three studies (57 rats) provided Tb.N data [10, 13, 14], and two studies (48 rats) provided Tb.Th and Tb.Sp data [13, 14]. In view of the significant heterogeneity (*I*^2^ > 50%, *P* < 0.1), we used a random effect model. There was a statistically significant difference on Tb.N between the BMSC transplantation group and the sham operation group based on the results of the pooled analysis (SMD = −6.84, 95% CI: [-12.37, -1.32], *P* = 0.015, *I*^2^ = 93.5%). However, there were no statistically significant differences on Tb.Th and Tb.Sp between the BMSC transplantation group and the sham operation group (Tb.Th: SMD = −2.95, 95% CI: [-8.1, 2.2], *P* = 0.261, *I*^2^ = 96.5%; Tb.Sp: SMD = 5.59, 95% CI: [-5.22, 16.41], *P* = 0.311, *I*^2^ = 97.7%).

### 3.5. Publication Bias

**Figure 9:**
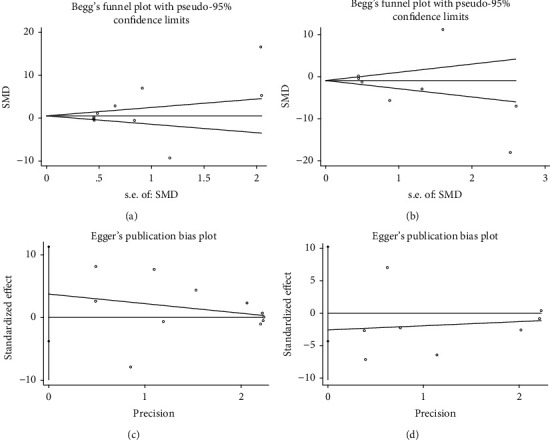
Assessment of publication bias in relevant studies that include Tb.N, Tb.Th, and Tb.Sp. Begg's test: (a) BMSCs vs. OVX control and (b) BMSCs vs. sham control; Egger's test: (c) BMSCs vs. OVX control and (d) BMSCs vs. sham control.

Publication bias is now commonly assessed in meta-analysis using Begg's funnel plot and Egger's test and is usually performed in at least 10 studies [24]. Because of the high heterogeneity in the results of the above pooled analysis, we performed an assessment of publication bias. Since *P* < 0.05 for Begg's test and Egger's test results, this suggests a possible publication bias for the included studies of BMD (stem cell treatment group vs. OVX control group: Begg's test: *P* < 0.001, Egger's test: *P* < 0.001; stem cell treatment group vs. sham operation group: Begg's test: *P* < 0.001, Egger's test: *P* = 0.001) and BV/TV (stem cell treatment group vs. OVX control group: Begg's test: *P* = 0.016, Egger's test: *P* = 0.001; stem cell treatment group vs. sham operation group: Begg's test: *P* = 0.027, Egger's test: *P* < 0.001). There was no publication bias in the studies included in the Tb.N, Tb.Th, and Tb.Sp because *P* > 0.05 for the results of Begg's test and Egger's test (BMSC transplantation group vs. OVX control group: Begg's test: *P* = 0.213, Egger's test: *P* = 0.289; BMSC transplantation group vs. sham operation group: Begg's test: *P* = 0.174, Egger's test: *P* = 0.44). We discussed the following reasons for the above conclusions: BMD and BV/TV are subgroup analyses based on different stem cell types, and the conditions of interventions are significantly different. Each stem cell type was explored with different characteristics, so the results of BMD and BV/TV showed a possible publication bias. In contrast, the analyses of Tb.N, Tb.Th, and Tb.Sp all belonged to one stem cell type, BMSCs, but were evaluated in different ways, so no publication bias was found.  Figure 9 shows the results of Begg's test and Egger's test assessing the publication bias of related studies that include Tb.N, Tb.Th, and Tb.Sp.

### 3.6. Sensitivity Analysis

**Figure 10:**
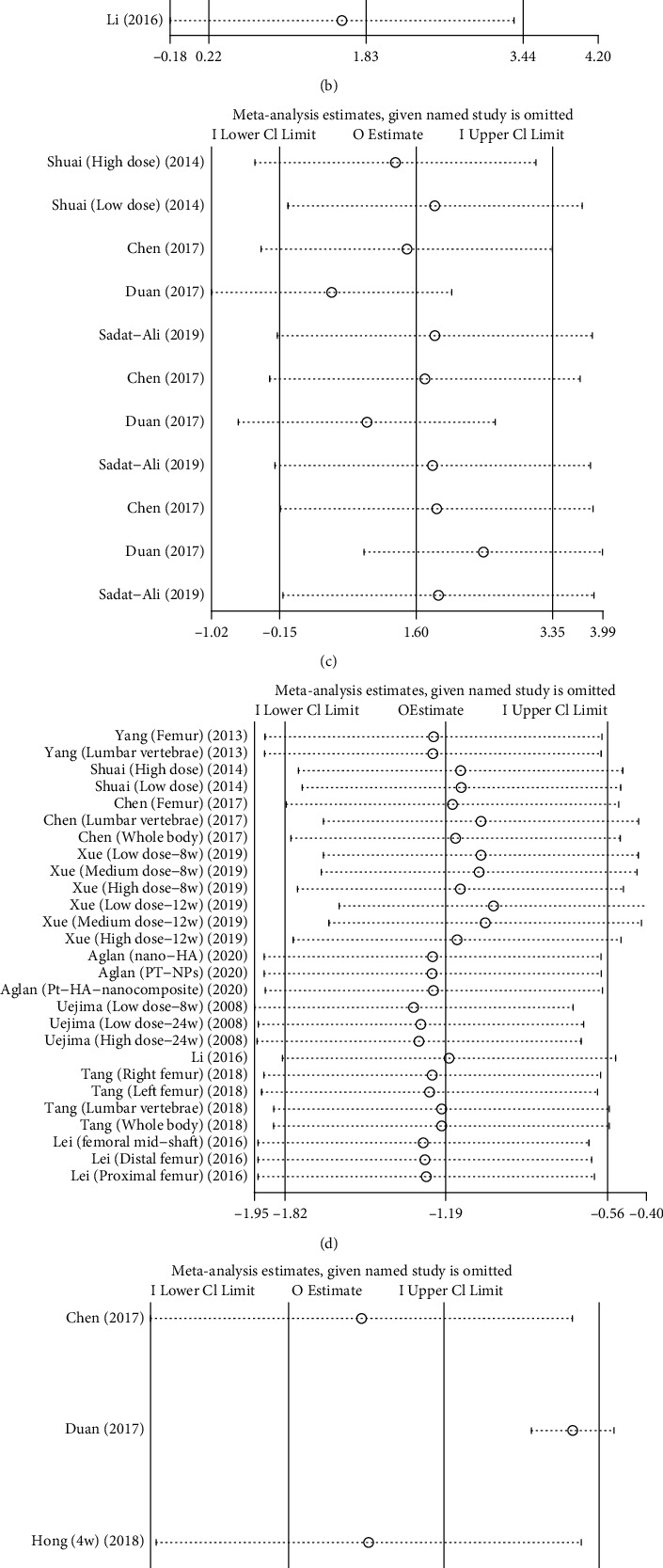
Sensitivity analysis of this meta-analysis. BMSCs vs. OVX control: (a) BMD, (b) BV/TV, and (c) Tb.N, Tb.Th, and Tb.Sp; BMSCs vs. sham control: (d) BMD, (e) BV/TV, (f) Tb.N, Tb.Th, and Tb.Sp.

Sensitivity analysis is also usually performed in a meta-analysis to assess the stability of the results of the pooled literature analysis [24]. We used a sensitivity analysis by removing all the included literature for all outcome measurements one by one and also used STATA software to plot the sensitivity analysis figures (Figure 10). Sensitivity analysis could not be effectively performed due to the limited number of relevant literature or less than 2 articles for some of the outcome indicators. We analyzed the outcome measurements for which the number of literature was sufficient, and no significant changes were found in their results, thus confirming the robustness and reliability of the results. Sources of the high heterogeneity in outcome measurements that emerged in this meta-analysis may be as follows: (1) different dosing regimens and timing of interventions in the included studies in each stem cell type, (2) limited number of studies included for the outcome measurements, (3) sample sizes and timing of collection of the outcome measurements were not identical in the included studies, and (4) inherent differences between studies.

## 4. Discussion

This meta-analysis explored the efficacy of stem cells in the treatment of osteoporosis. Osteoporosis is defined as a systemic bone disease characterized by decreased bone mass and deterioration of bone microstructure, resulting in increased bone fragility and prone to fractures [7]. As a type of cell with unlimited proliferation and differentiation potential, stem cells have been used in the treatment and prevention of a variety of complex diseases [17]. Currently, stem cell transplantation is mostly used in animal models of osteoporosis, including rats, mice, rabbits, experimental monkeys, and pigs. The establishment of animal models of osteoporosis is mostly achieved by ovariectomy. The commonly used stem cells include BMSCs, ADSCs, AM-MSCs, and hUCB-MSCs [9–21]. Uejima et al. [21] demonstrated that direct BMSC injection may improve bone strength. Uri et al. [16] found that the implantation of autologous ADSCs into the proximal femur of OVX osteoporotic rats could promote bone regeneration and enhance bone strength during short-term follow-up. Lei et al. [12] demonstrated that the synergistic application of AM-MSCs and zoledronic acid could improve the symptoms of OVX osteoporosis rats. Hong et al. [20] found that hUCB-MSCs could enhance bone regeneration in a rat model of osteoporosis. Elseweidy et al. [26] demonstrated that the combination of MSC and the antioxidant resveratrol was more effective in increasing bone mass and improving osteoporosis than treatment alone. Some studies had shown that the combination of platelet-rich fibrin releasates (PRFr) and BMSCs [27] or ADSCs [28] could reduce bone loss in OVX osteoporotic mice. Based on the above findings, we can find that stem cell transplantation holds promise as a potential treatment for osteoporosis.

Thirteen articles that met the inclusion criteria were included in this meta-analysis, seven of which were written in English [13, 15–17, 19–21] and the remaining six were written in Chinese [9–12, 14, 18]. The 13 studies included four different sources of stem cells, and the osteoporosis models were all constructed by ovariectomy [9–21]. The control group included two types, one for the osteoporosis model constructed successfully but without stem cell transplantation (OVX control) and the other for the osteoporosis model not constructed but with skin incision (sham operation). Outcome measurement obtained based on micro-CT and dual-energy X-ray absorptiometry included BMD, BV/TV, Tb.N, Tb.Th, and Tb.Sp [9–21]. BMD, an important indicator of bone strength; BV/TV, a histomorphometric parameter used to measure bone strength; and Tb.Th, Tb.Sp, and Tb.N, standard parameters used to investigate structural changes in bone trabeculae, are common analytical indicators to evaluate the effectiveness of osteoporosis treatment [29]. Therefore, because of the differences in the control groups, a pooled analysis was performed for both scenarios for these outcome measurements.

For BMD, the primary outcome measurement, BMD, was higher in the BMSC and ADSC transplantation groups when compared to the OVX control group, with no significant difference in the AM-MSC transplantation group; BMD was lower in the BMSC, ADSC, and AM-MSC transplantation groups when compared to the sham-operated group. The higher the BMD, the higher the strength of the bone [6]. Therefore, based on the results after this pooled analysis, we hypothesized that stem cell transplantation could increase bone strength in OVX osteoporotic rats, and the increased strength was lower than that in normal rats (sham-operated group). For BV/TV, a secondary outcome measurement, BV/TV, was higher in the hUCB-MSC and ADSC transplant groups when compared with OVX controls; when compared with the sham-operated group, BV/TV was lower in the hUCB-MSC transplant group and not significantly different in the BMSC transplant group, and ADSC could not be compared due to the limited number of studies. A higher BV/TV represents a relatively higher bone volume in the tissue [6]. Therefore, based on the results after this pooled analysis, we hypothesized that stem cell transplantation might increase bone volume in OVX osteoporotic rats, and the increased bone volume was lower than that in normal rats (sham-operated group). For Tb.N, Tb.Th, and Tb.Sp, a secondary outcome measurement, Tb.N, was higher in the BMSC transplantation group when compared with the OVX control group, while Tb.Th and Tb.Sp were not significantly different; Tb.N was lower in the BMSC transplantation group when compared with the sham-operated group, while Tb.Th and Tb.Sp were not significantly different. A higher Tb.N represents a relatively higher number of bone trabeculae in the tissue [6]. Therefore, based on the results after this pooled analysis, we hypothesized that stem cell transplantation could increase the number of bone trabeculae in OVX osteoporotic rats, and the increased number of bone trabeculae was lower than that in normal rats (sham-operated group). Moreover, the results of Tb.Th and Tb.Sp were not significant due to the relatively limited number of studies. The results of the pooled analysis suggest that increasing bone strength and bone mass, as well as the number of trabeculae, may make stem cell transplantation a potential treatment modality for osteoporosis. However, because the therapeutic effect varies with different sources of stem cells, there is still a need to explore more deeply the effect of different stem cells for the treatment of osteoporosis in the future. The details explored include which stem cells are the best choice, as well as the dose, timing, and manner of use.

We summarized the original results of the included studies and presented the results of the group comparisons between the BMSC group and the OVX control group and sham control group on BMD in a table (Table 3). As can be seen from the raw results above, data for the same outcome measurements from different studies were pooled, meta-analysis was performed, and the results obtained were generally consistent with the raw results. It also shows the credibility of the results of the meta-analysis. However, for subgroup analyses containing only one study, the results still need to be supplemented by additional studies in the future. The results of this meta-analysis were analyzed by pooling data from several related studies, expanding the sample size, and yielding results that are generally consistent with individual data, providing direction for the next step in conducting related studies. Therefore, this article is necessary and the results are relatively reliable.

It is well known that cancer cells and stem cells share a remarkable commonality of continuous self-replication, and the molecular mechanisms remain a hot topic of research for scientists worldwide [30]. Therefore, there is also a risk of developing cancer cells after stem cell transplantation. Along with the possibility of recurrence of the original cancer after treatment with stem cell transplantation, there is also the possibility of a second cancer developing after transplantation [31]. Studies have shown that people who have received allogeneic hematopoietic stem cell transplantation are at higher risk of developing a second cancer [32]. Other types of stem cells are also at risk of developing into cancer cells, influenced by the tumor microenvironment in which they are embedded [33]. Therefore, stem cell transplantation also has two sides to it, and it is important to recognize the beneficial effects it has in treating diseases, but also not to ignore the potential side effects, such as the risk of cancer.

### 4.1. Limitations

Due to the number and quality of included studies, this meta-analysis has certain limitations. First, the number of RCTs included is relatively limited, and some studies are of relatively low quality. Secondly, in different studies, the dosage, time, and method of using the same type of stem cells are different. Third, the heterogeneity of some results is high. Fourth, the type of rats, the age of the month, the average weight, and the construction time of the osteoporosis model are not completely consistent. Finally, the number of studies on some outcome measurements is limited, and effective results cannot be obtained.

## 5. Conclusion

This is a meta-analysis based on the results of micro-CT and dual-energy X-ray absorptiometry to evaluate the effect of stem cell transplantation on OVX osteoporotic rats. The results of the above analysis suggest that stem cell transplantation is a potential therapeutic direction for osteoporosis by possibly increasing bone strength, bone volume, and the number of trabeculae in OVX osteoporotic rats. However, the levels of these outcome measurements after the increase were lower than those of normal rats (sham-operated group). Due to the limited number and quality of studies related to this, more high-quality RCTs are still needed in the future to complement the existing findings.

## Figures and Tables

**Table 1:** Characteristics of all studies included in the meta-analysis.

Study	Country	Study type	Animal models	Age and weight	No. of rats	Osteoporosis model	Experimental group	Injection dose	Control group	Detection time^∗^	Detection sites	Outcome measures
Uejima et al. (2008)	Japan	Non-RCT	SD, female, rats	12 w	10/10	OVX	BMSCs	1 × 10^5^ 1 × 10^5^ 2 × 10^5^	Sham-operation+saline	8 w 24 w 24 w	Femur	(1)
Yang et al. (2013)	China	RCT	SD, female, rats	230 ± 10 g	10/10/10	OVX	BMSCs	3 × 10^6^	OVX control, sham-operation	12 w	Femur, lumbar vertebrae	(1)
Shuai et al. (2014)	China	RCT	SD, female, rats	140-150 g	4/4/4	OVX	BMSCs	1.5 × 10^7^ 0.375 × 10^7^	OVX control, sham-operation+PBS	10 w 10 w	Femur	(1), (3)
Lei et al. (2016)	China	RCT	Wistar, female, rats	210-255 g	15/15/45	OVX	AM-MSCs+zoledronic acid	NP	OVX control, sham-operation	13 w	Femur	(1)
Li et al. (2016)	China	RCT	SD, female, rats	7 w, 180-220 g	10/10/10	OVX	ADSCs	2 × 10^6^	OVX control, sham-operation+PBS	6 w	Femur	(1), (2)
Chen et al. (2017)	China	RCT	SD, female, rats	7 w, 180-220 g	10/10/10	OVX	BMSCs	3.5 × 10^9^	OVX control, sham-operation+PBS	2 w	Femur, lumbar vertebrae	(1), (2), (3)
Duan et al. (2017)	China	RCT	SD, female, rats	10 w, 110 ± 8.73 g	18/10	OVX	BMSCs	2 × 10^5^	OVX control, sham-operation	8 w	Tibia	(2), (3)
Hong et al. (2018)	South Korea	RCT	SD, female, rats	12 w	10/10/10	OVX	hUCB-MSCs	1 × 10^7^ 1 × 10^7^	OVX control, sham-operation	4 w 8 w	Femur	(3)
Tang et al. (2018)	China	RCT	SD, female, rats	3 m, 250 ± 20 g	10/10/10	OVX	ADSCs+icariin	NP	OVX control, sham-operation	12 w	Femur, lumbar vertebrae	(1)
Uri et al. (2018)	Israel	RCT	SD, female, rats	5 m, 260 − 320 g	15/15	OVX	ADSCs	NP	OVX control	NP	Femur	(3)
Xue et al. (2019)	China	RCT	SD, female, rats	205 ± 11 g	30/10/10	OVX	BMSCs	2 × 10^6^ 4 × 10^6^ 6 × 10^6^	OVX control, sham-operation	4 w, 8 w 4 w, 8 w 4 w, 8 w	Tibia	(1)
Sadat-Ali et al. (2019)	Saudi Arabia	Non-RCT	SD, female, rats	NP	10/10	OVX	BMSCs	2 × 10^6^	OVX control+saline	8 w	Femur	(2), (3)
Aglan et al. (2020)	Egypt	Non-RCT	Wistar, female, rats	130-150 g	30/10/10	OVX	BMSCs+nano-HA BMSCs+Pt-NPs BMSCs+Pt-HA-nanocomposite	3 × 10^6^	OVX control, sham-operation	2 m	Femur	(1)

RCT: randomized controlled trial; SD: Sprague Dawley; OVX: ovariectomized; BMSCs: bone marrow mesenchymal stem cells; ADSCs: adipose-derived stem cells; AM-MSCs: amniotic membrane mesenchymal stem cells; hUCB-MSCs: human umbilical cord blood-derived mesenchymal stem cells; PBS: phosphate-buffered saline; NP: not provided. Outcome measures: (1): BMD; (2): BV/TV; (3): Tb.N, Tb.Th, and Tb.Sp. ^∗^Detection time: from after stem cell injection.

**Table 2:** The Newcastle-Ottawa Scale (NOS) for assessing the quality of non-RCTs.

Study	Selection	Comparability	Outcomes	Total scores (maximum 9)
Uejima et al. [21]	3	1	2	6
Sadat-Ali et al. [17]	3	1	2	6
Aglan et al. [19]	3	2	2	7

**Table 3:** Basic information about the included studies containing BMD.

Study	Stem cell transplantation	OVX control	Sham control	Unit of measurement	Stem cell type	Subgroup
Yang et al. [9]	0.1797 ± 0.0292^∗^^#^	0.1539 ± 0.0745	0.189 ± 0.0461	g/cm^2^	BMCs	Femur
Yang et al. [9]	0.1607 ± 0.0893^∗^^#^	0.1396 ± 0.0631	0.1742 ± 0.0796	g/cm^2^	BMCs	Lumbar vertebrae
Xue et al. [18]	0.173 ± 0.0027^∗^^#^	0.165 ± 0.0033	0.187 ± 0.0033	g/cm^2^	BMCs	Low dose-8 w
Xue et al. [18]	0.173 ± 0.0031^∗^^#^	0.165 ± 0.0033	0.187 ± 0.0033	g/cm^2^	BMCs	Medium dose-8 w
Xue et al. [18]	0.178 ± 0.004^∗^^#^	0.165 ± 0.0033	0.187 ± 0.0033	g/cm^2^	BMCs	High dose-8 w
Xue et al. [18]	0.165 ± 0.0026^∗^^#^	0.158 ± 0.0047	0.185 ± 0.0028	g/cm^2^	BMCs	Low dose-12 w
Xue et al. [18]	0.169 ± 0.0032^∗^^#^	0.158 ± 0.0047	0.185 ± 0.0028	g/cm^2^	BMCs	Medium dose-12 w
Xue et al. [18]	0.178 ± 0.0036^∗^^#^	0.158 ± 0.0047	0.185 ± 0.0028	g/cm^2^	BMCs	High dose-12 w
Shuai et al. [10]	361 ± 21^∗^^#^	250 ± 6	431 ± 20	mg/cm^3^	BMCs	High dose
Shuai et al. [10]	253 ± 6^#^	250 ± 6	431 ± 20	mg/cm^3^	BMCs	Low dose
Chen et al. [14]	206.29 ± 19.55^∗^^#^	193.98 ± 25.38	243.31 ± 20.11	mg/cm^2^	BMCs	Femur
Chen et al. [14]	126.11 ± 12.69^∗^^#^	102.21 ± 11.56	180.32 ± 10.55	mg/cm^2^	BMCs	Lumbar vertebrae
Chen et al. [14]	110.33 ± 10.21^∗^^#^	101.21 ± 8.75	130.21 ± 9.21	mg/cm^2^	BMCs	Whole body
Aglan et al. [19]	191.6 ± 26.9^∗^	147.7 ± 8.7	194.9 ± 16.9	mg/cm^2^	BMCs	Nano-HA
Aglan et al. [19]	191.5 ± 27^∗^	147.7 ± 8.7	194.9 ± 16.9	mg/cm^2^	BMCs	Pt-NPs
Aglan et al. [19]	190.5 ± 15.7^∗^	147.7 ± 8.7	194.9 ± 16.9	mg/cm^2^	BMCs	Pt-HA-nanocomposite
Uejima et al. [21]	139.35 ± 4.63^#^	NP	124.32 ± 4.88	mg/cm^2^	BMCs	Low dose-8 w
Uejima et al. [21]	154.65 ± 5.1^#^	NP	147.42 ± 4.36	mg/cm^2^	BMCs	Low dose-24 w
Uejima et al. [21]	149.89 ± 10.37^#^	NP	133.42 ± 7.37	mg/cm^2^	BMCs	High dose-24 w

OVX: ovariectomized; BMSCs: bone marrow mesenchymal stem cells; NP: not provided. ^∗^Significant change at *P* < 0.05 in comparison with the OVX control group. ^#^Significant change at *P* < 0.05 in comparison with the sham control group.

## Data Availability

The data supporting this meta-analysis is from previously reported studies and datasets, which have been cited.
